# To Cure Drunkenness

**Published:** 1889-09

**Authors:** 


### TO CURE DRUNKENNESS.

A Russian physician named Portugaloff declares that strychnine is an infallible cure for drunkenness, administered in subcutaneous injection. He asserts that the experience of physicians has shown the cure to be as rapid as it is certain. The effect of the strychnine solution is to change the craving for drink into positive aversion, and this change is effected in a day. After a treatment of eight or ten days a patient may be discharged. The strychnine is administered by dissolving one grain in 200 drops of water and injecting five drops of the solution every twenty-four hours.

